# Supplementary material for: Christensenella minuta Alleviates Acetaminophen-Induced Hepatotoxicity by Regulating Phenylalanine Metabolism
Source: Nutrients. 2024 Jul 18;16(14):2314. doi: 10.3390/nu16142314 (PMC11280030; doi:10.3390/nu16142314)
Supplement: Supplementary file 1 [file nutrients-16-02314-s001.zip › nutrients-3063128-supplementary.pdf]

Table S1. Oligonucleotide sequences used for Q-PCR analysis.

Species	Primer	Sequences (5'-3')
Human	IL10	F 5’- GACTTTAAGGGTTACCTGGGTTG -3’
		R 5’- TCACATGCGCCTTGATGTCTG -3’
	GAPDH	R 5’-CTGGGCTACACTGAGCACC-3’
		F 5’-AAGTGGTCGTTGAGGGCAATG-3’
Mouse	Tnfα	F 5’- CAGGCGGTGCCTATGTCTC -3’
		R 5’- CGATCACCCCGAAGTTCAGTAG -3’
	Il1β	F 5’- ATGATGGCTTATTACAGTGGCAA -3’
		R 5’- GTCGGAGATTCGTAGCTGGA -3’
	Il6	F 5’- CTGCAAGAGACTTCCATCCAG -3’
		R 5’- AGTGGTATAGACAGGTCTGTTGG -3’
	Igf1	F 5’- GTCGTCTTCACACCTCTTCTACCT -3’
		R 5’- GCACAGTACATCTCCAGTCTCCT -3’
	Igf1r	F 5’- CTTCTACAACTACGCACTGGTC -3’
		R 5’- TCGGCGTTCTTCTCAATCCTG -3’
	Gapdh	F 5’-AGGTCGGTGTGAACGGATTTG-3’
		R 5’-GGGGTCGTTGATGGCAACA-3’

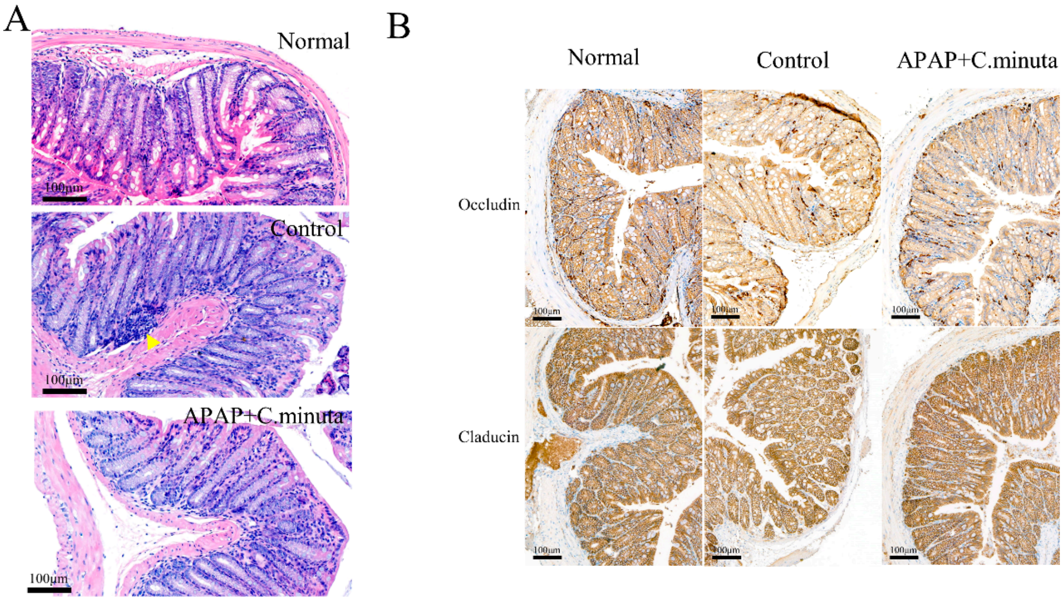

Figure S1. (A) Representative images of H&E staining in mouse colon (scale bar = 100 μm, yellow arrow: immunocytes) (B) Representative images of H&E staining of the expression of Occludin and Claducin (scale bar = 100 μm).

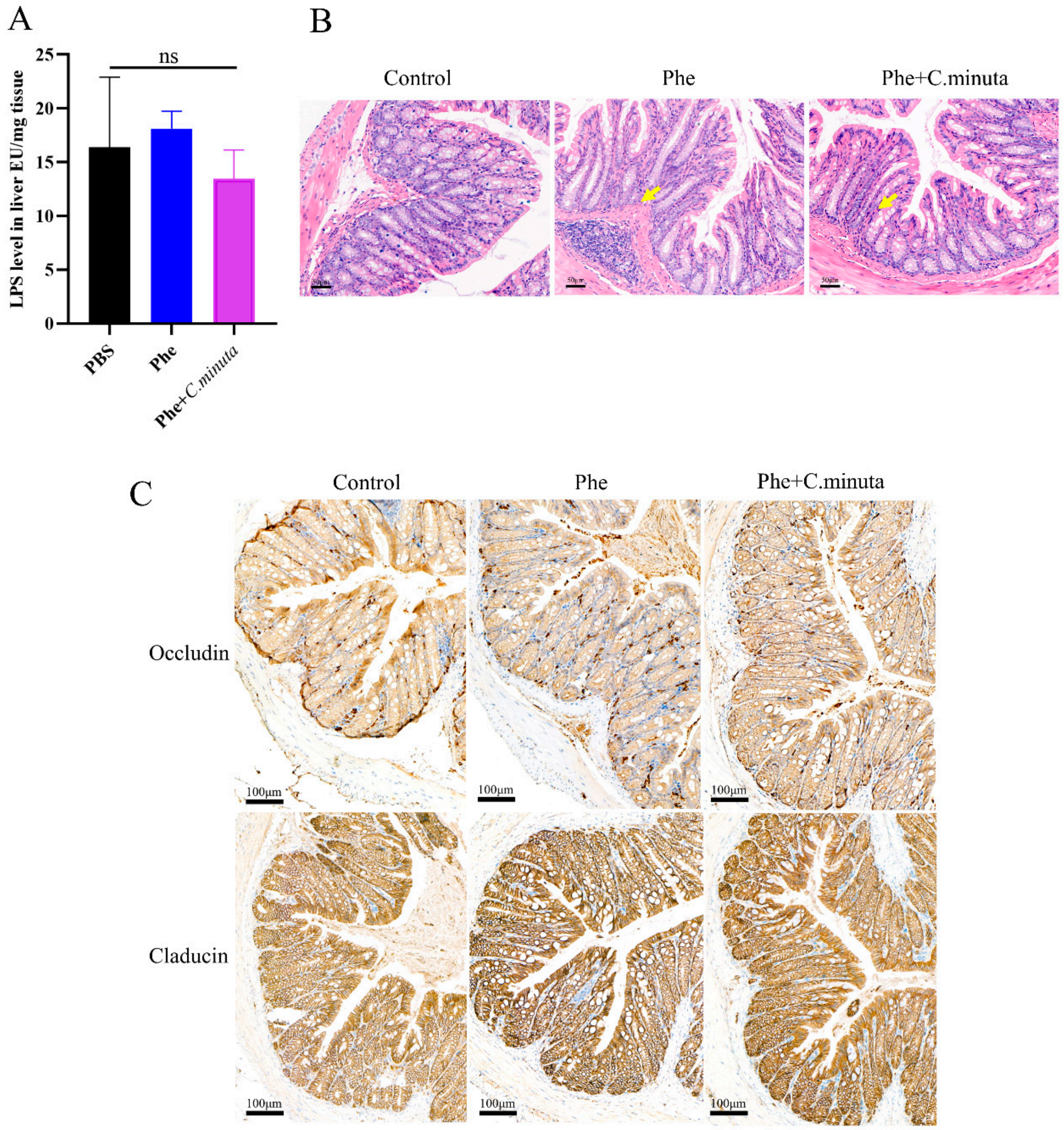

Figure S2. (A) The LPS level in mice liver measured by EILSA. (B) Representative images of H&E staining in mouse colon (scale bar = 100 μm, yellow arrow: immunocytes) (C) Representative images of H&E staining of the expression of Occludin and Claducin in mouse colon (scale bar = 100 μm).

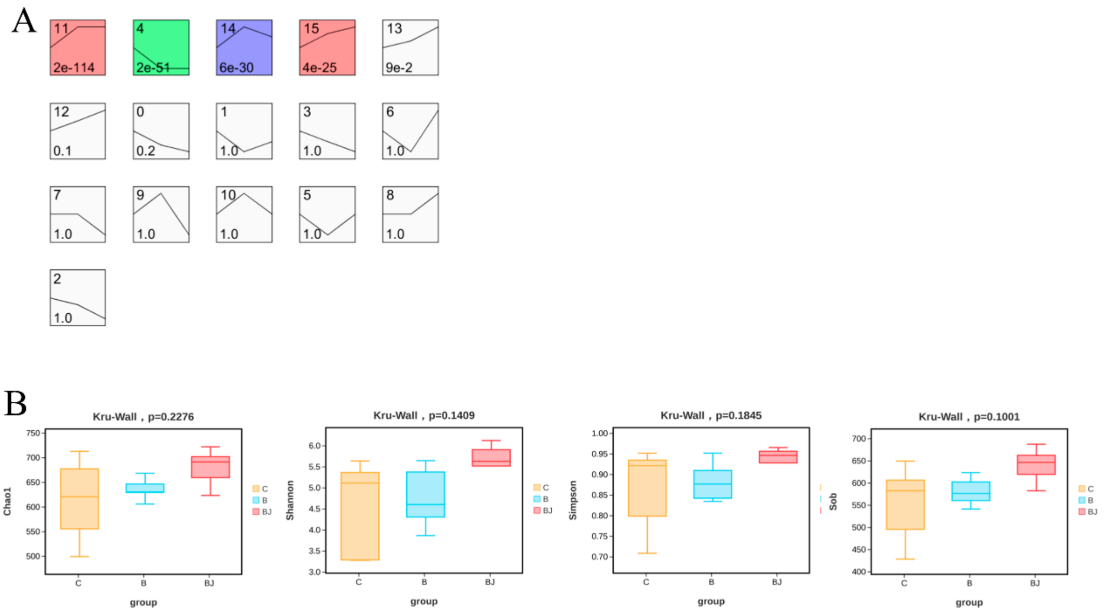

Figure S3. (A) the Trends in gene expression changes analyzed by STEM analysis. (B) Abundance and alpha diversity of microbial (Shannon, Simpson, Chao, and Sob) in Control, Phe, Phe+C.minuta group. C represented the Control group, B represented Phe group, BJ represented Phe+C.minuta group.
